# Supplementary material for: Effect of thermal gradients on inhomogeneous degradation in lithium-ion batteries
Source: Commun Eng. 2023 Oct 21;2:74. doi: 10.1038/s44172-023-00124-w (PMC10956044; doi:10.1038/s44172-023-00124-w)
Supplement: Supplementary file 2 — Supplementary material [file 44172_2023_124_MOESM2_ESM.pdf]

Supplementary Material

Inhomogeneous degradation in lithium-ion batteries: the effect of thermal gradients

Shen Li[1,4, §], Cheng Zhang[2], Yan Zhao[3], Gregory J. Offer[1,4], Monica Marinescu[1,4, *]

* Corresponding author.

E-mail address: monica.marinescu@imperial.ac.uk

Affiliations:

1. **Department of Mechanical Engineering, Imperial College London, London SW7 2AZ, United Kingdom**
2. **Institute for Future Transport and Cities, Coventry University, Coventry CV1 5FB, United Kingdom**
3. **Breathe Battery Technologies Limited, London SE1 7SJ, United Kingdom**
4. **The Faraday Institution, Harwell Science and Innovation Campus, Didcot OX11 0RA, United Kingdom**

§ Present address: Rimac Technology R&D UK Limited, Warwick CV35 9EF, United Kingdom.

Supplementary Note 1: Convergence check on the ECN model

A convergence study was performed to assess whether the configured mesh in the distributed ECN model is adequate. The temperature at cell core (i.e. the centre ECN unit in both length and thickness direction, as the probable place for the highest error) was compared while different meshing schemes were applied. The cell was discharged at a constant current of 6C under surface cooling scenario, which is more aggressive than the tab cooling scenario in generating a thermal gradient within the cell. With the normal mesh, the whole cell is divided by 45 electrical/thermal ECN units: 3 units x 3 units along length x width, and 5 units along thickness. With a denser mesh, 1377 units are used: 9 x 9 x 17 units along length, width and thickness direction. **Fig. S1** shows the cell core temperature calculated under two meshing schemes. The final temperature changes by 0.6%, value considered too low to justify a 30-fold increase in mesh density, especially when considering the constraints on the speed of the model for long-term cycling degradation. The normal mesh scheme was applied.

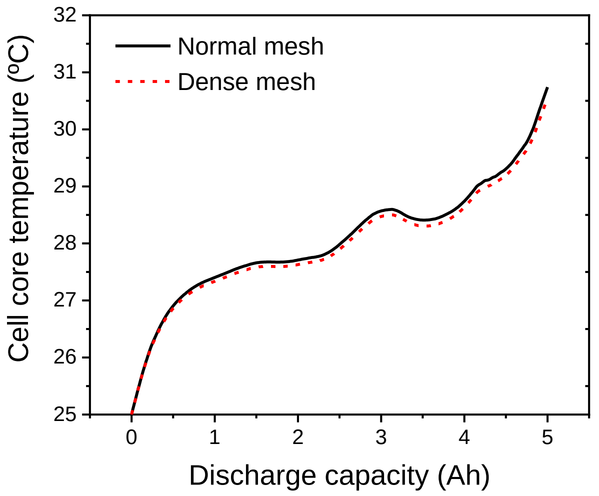

Fig. S1. Comparison of cell core temperature for distributed electro-thermal ECN model with the normal mesh and denser mesh.

Supplementary Note 2: Degradation function modification

The resistance increase rate given by equation (12) is plotted in **Fig. S2**(a). Due to the cell-level parametrization problem discussed in **Fig. 4** in the main text, the current dependency of the rate of resistance increase is weaker than that of the real degradation function. As the degradation function parametrized from experimental data ignores the fact that cells have probably undergone inhomogeneous degradation, the fitted function is not suitable to be used in the inhomogeneity-aware distributed model. The experimentally parametrized function by Cordoba-Arenas et al [1] is adapted here to result in a 'current-downward' (i.e. the rate of resistance decreases when the current increases) current dependency by a tuning parameter n_0:

$$\frac{\mathrm{d}\mu}{\mathrm{d}w} = \left\{556.08 \cdot \exp[-n_0(CR-2)] + 3219.81\right\} \cdot \exp\left(\frac{-51800}{8.32T}\right) \cdot V_0, \tag{S1}$$

where V_0 is the volume of material corresponding to one ECN unit, T is its temperature and CR the local C rate. The current dependency of the rate of resistance increase is magnified by the compression factor n_0 when $n_0 > 1$. This adapted degradation function is plotted in **Fig. S2**(b) for $n_0 = 1$ (no current dependency magnification), and has good agreement with the original law with RMSE of 4.51×10^{-8} (m^3/Ah). For the long-term simulation tests in the main text, $n_0 = 7.4$ is used; the resulting function is shown in **Fig. S2**(c).

As discussed in the Section 3.2 in the main text, a current-upward degradation law (i.e. the rate of resistance increases with current) may be present in some cases, subject to the complex interplay of degradation mechanisms. For a thorough study, this type of degradation law is also implemented for degradation simulation. The current-upward function is formed in

a symmetric way to the current-downward function, and the current-upward law is expressed as:

$$\frac{\mathrm{d}\mu}{\mathrm{d}w} = \left\{556.08 \cdot \exp[-n_0(CR+6)] + 3219.81\right\} \cdot \exp\left(\frac{-51800}{8.32T}\right) \cdot V_0 \, . \tag{S2}$$

The current-upward function when n_0 = 4.0 is plotted in **Fig. S2**(d) and this function is used in the simulation in Supplementary Note 3.

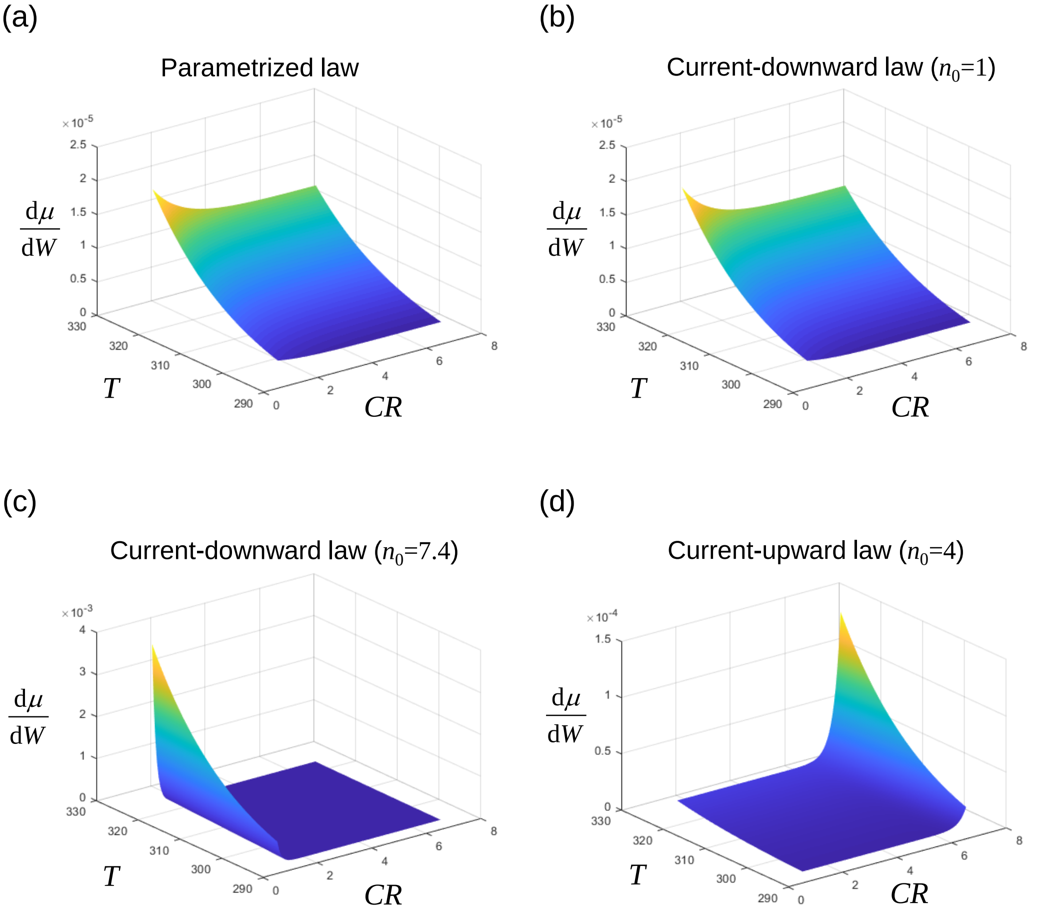

Fig. S2. Degradation functions. (a) Parametrized ageing law of resistance increase from reference[1]. In the original reference, *Ratio* and SoC_{min} are additional variables for charge-depleting and charge-sustaining ratio and cycling SoC lower limit. They are set to be 1 and 0.25 for use in this study. (b) Current-downward law when n_0 = 1. (c) Current-downward law when n_0 = 7.4. (d) Current-upward law when n_0 = 4.0.

Supplementary Note 3: Cycling degradation under cooling strategies for current-upward resistance law

Cycling degradation using another ageing law (i.e. current-upward law) was also performed for the surface and tab cooling schemes. The same simulation setup is used as for **Fig. 7** in the main text, with the sole difference in the resistance increase law used for the electrical-thermal-degradation model, given now by equation (S2). As in the case of current-upward ageing law, the simulation results of cycling degradation predict that the available capacity loss is significantly faster for surface cooling than tab cooling, as shown in **Fig. S3**(a). The rate of loss of available capacity under the two cooling scenarios is compared in **Fig. S3**(b) for both experimental data and simulation results.

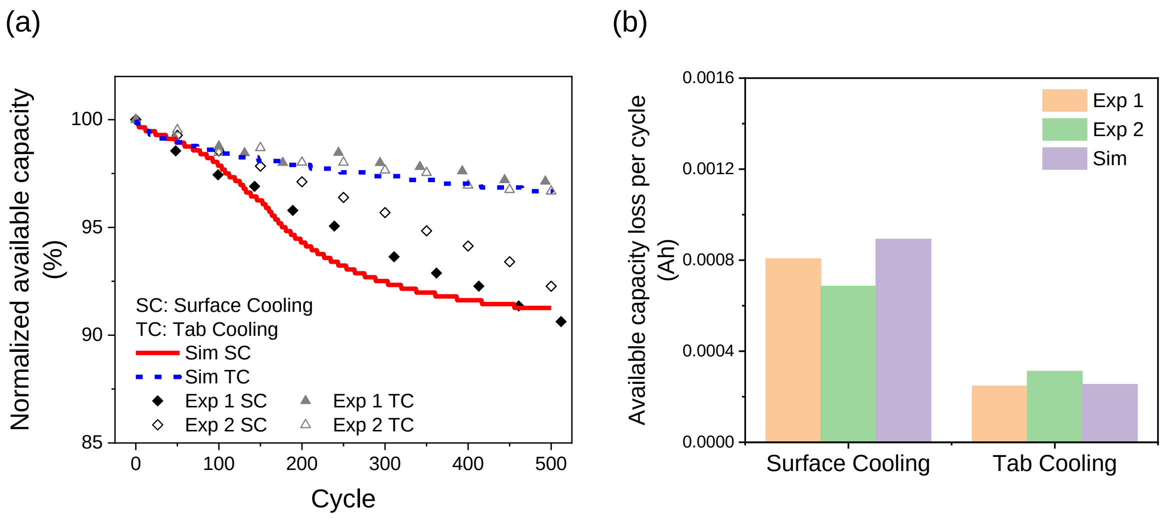

Fig. S3. Cycling degradation simulation using current upward law for the surface and tab cooling schemes. (a) Comparison of simulation results and experimental data retrieved from Hunt et al[2] for normalized available capacity. The blue dashed and red solid lines represent the results simulated under the surface and tab cooling schemes, respectively. The diamonds and triangles symbols represent the experimental data under the surface and tab cooling schemes, respectively. (b) The rate of loss of available capacity for the two cooling schemes.

The orange and green bars represent the experimental data, including one repeat. The purple bars represent the simulation results.

To identify the underlying mechanisms for the accelerated degradation under the surface cooling scheme, the same analysis was conducted as following **Fig. 7** in the main text; the ensuing internal inhomogeneities of current, resistance increase and temperature are shown in **Fig. S4.** The non-uniform currents are initialized in the first cycles (as shown in upper inset of **Fig. S4**(a)) and the currents become significantly divergent during the long-term cycling (as shown in lower inset of **Fig. S4**(a)). Meanwhile, due to the interaction between inhomogeneities of current and resistance, the non-uniform resistance increase is magnified through the cycles as shown in **Fig. S4**(c). In contrast to the more uniform currents (**Fig. S4**(b)) and resistance increase (**Fig. S4**(c)) for tab cooling, the significant inhomogeneities in the surface-cooled cell are attributed to the thermal gradients (ΔT) generated by the two cooling schemes, as shown in upper insets of **Fig. S4**(e) and (f). Tab cooling generates higher T_{avg} and causes faster fundamental capacity loss (as shown in **Fig. S4**(g) and (h)). However, in this simulation case, the available capacity loss is dominated by the temperature inhomogeneities (ΔT) rather than the magnitude of the average temperature (T_{avg}).

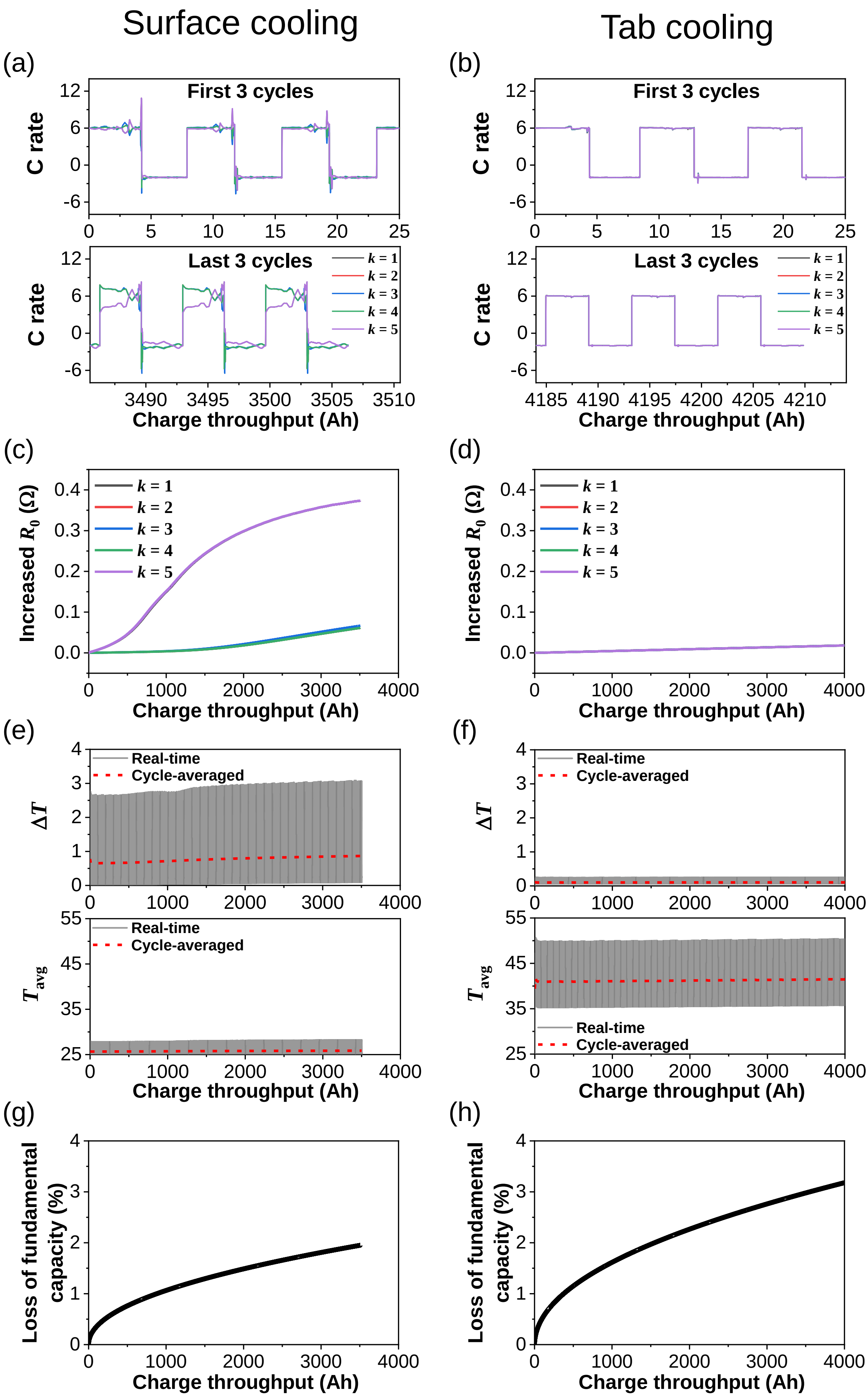

Surface cooling
Tab cooling
(a)
(b)
First 3 cycles
Last 3 cycles
C rate
k = 1
k = 2
k = 3
k = 4
k = 5
Charge throughput (Ah)
(c)
(d)
Increased R_0 (Ω)
(e)
(f)
Real-time
Cycle-averaged
ΔT
T_{avg}
(g)
(h)
Loss of fundamental capacity (%)

Fig. S4. Comparison of degradation behavior under surface and tab cooling approaches. C rate distribution for the five ECN units along the center line in cell thickness direction: (a) surface cooling and (b) tab cooling scheme. The upper inset shows the first 3 cycles and the lower inset shows the last three cycles. The history-dependent increase to R_0 is shown for the ECN units for (c) surface cooling and (d) tab cooling scheme. The variables for units k =1 to k = 4 in (c) are overlapping. Thermal gradient ΔT and average temperature $\underline{T}_{avg}$ are shown for (e) surface cooling and (f) tab cooling scheme. The grey lines represent the real-time data and the red dotted curves represent the cycle-averaged value. The loss of fundamental capacity percentage for the whole cell is shown for (g) surface cooling and (h) tab cooling scheme.

Supplementary Note 4: Cycling degradation using resistance laws with no and weak current dependency

A cycling degradation study was performed for the case of weak and no current dependency in the ageing law, under surface cooling. The simulation setup is the same as for **Fig. 6** in the main text, with the only difference being the resistance increase law. For the degradation function with no current dependency, the current dependency in equation (S1) is eliminated by fixing $CR = 2$. The degradation function is then only temperature dependent and given as:

$$\frac{\mathrm{d}\mu}{\mathrm{d}w} = 3775.89 \cdot \exp\left(\frac{-51800}{8.32T}\right) \cdot V_0 . \tag{S3}$$

For the degradation function with weak current dependency, the compression factor n_0 describing the current sensitivity in equation (S1) is set as $n_0 = 1$.

As shown in **Fig. S5**(a) and (b), the rates of loss of available capacity in the two studies are similar and underestimate the rate observed in the experimental results, unlike the previous simulation results using degradation functions with strong current dependency. These results indicate that a strong C rate dependency in the degradation function is a trademark of degradation, at least in these cells.

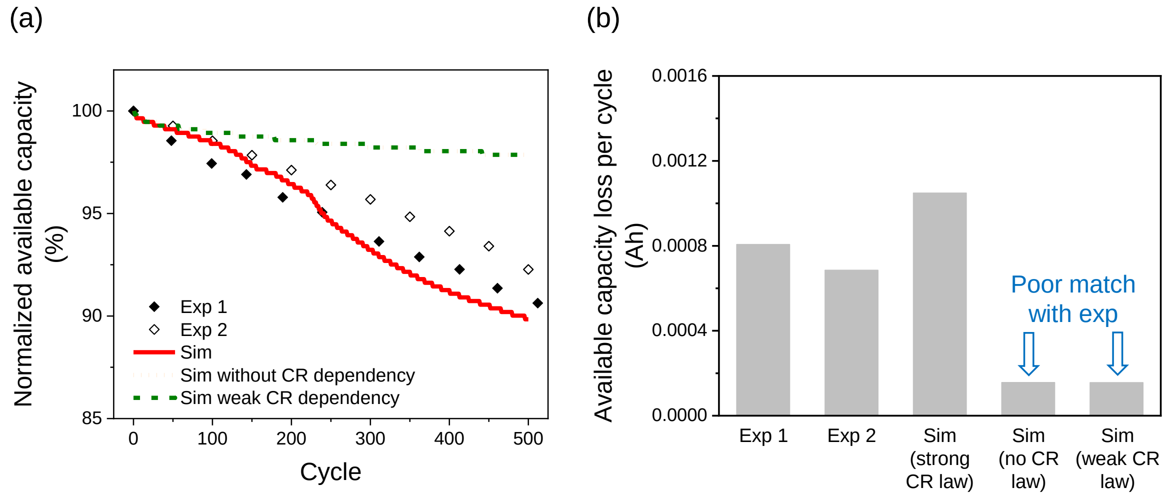

Fig. S5. Cycling degradation simulation using no current dependency and weak current dependency for surface cooling. (a) Comparison of simulation results using a resistance law with high and low current dependency and without current dependency for surface cooling. Surface cooling experimental data are retrieved from Hunt et al[2]. The yellow dotted line and green dashed line represent the simulated results for no C rate dependency and low C rate dependency, respectively. The diamonds represent the experimental data under surface cooling. (b) Rate of loss of available capacity for the surface cooling scheme.

The cell internal inhomogeneities are analyzed. The current inhomogeneities are apparent in the first 3 cycles, as shown in upper panel of **Fig. S6**(a) and (b) for the two runs. Under surface cooling, those current inhomogeneities are generated by the internal thermal gradients, as shown in the upper panel in **Fig. S6**(e) and (f). In contrast to the CR-sensitive degradation results (as shown in **Fig. 7** (a) and (c) in the main text), here the inhomogeneities of current (lower panel of **Fig. S6**(a) and (b)) and resistance (**Fig. S6**(c) and (d)) do not increase with cycling. In this case, in which the current dependency of the degradation law is reduced, no positive feedback between inhomogeneities of current and resistance is generated.

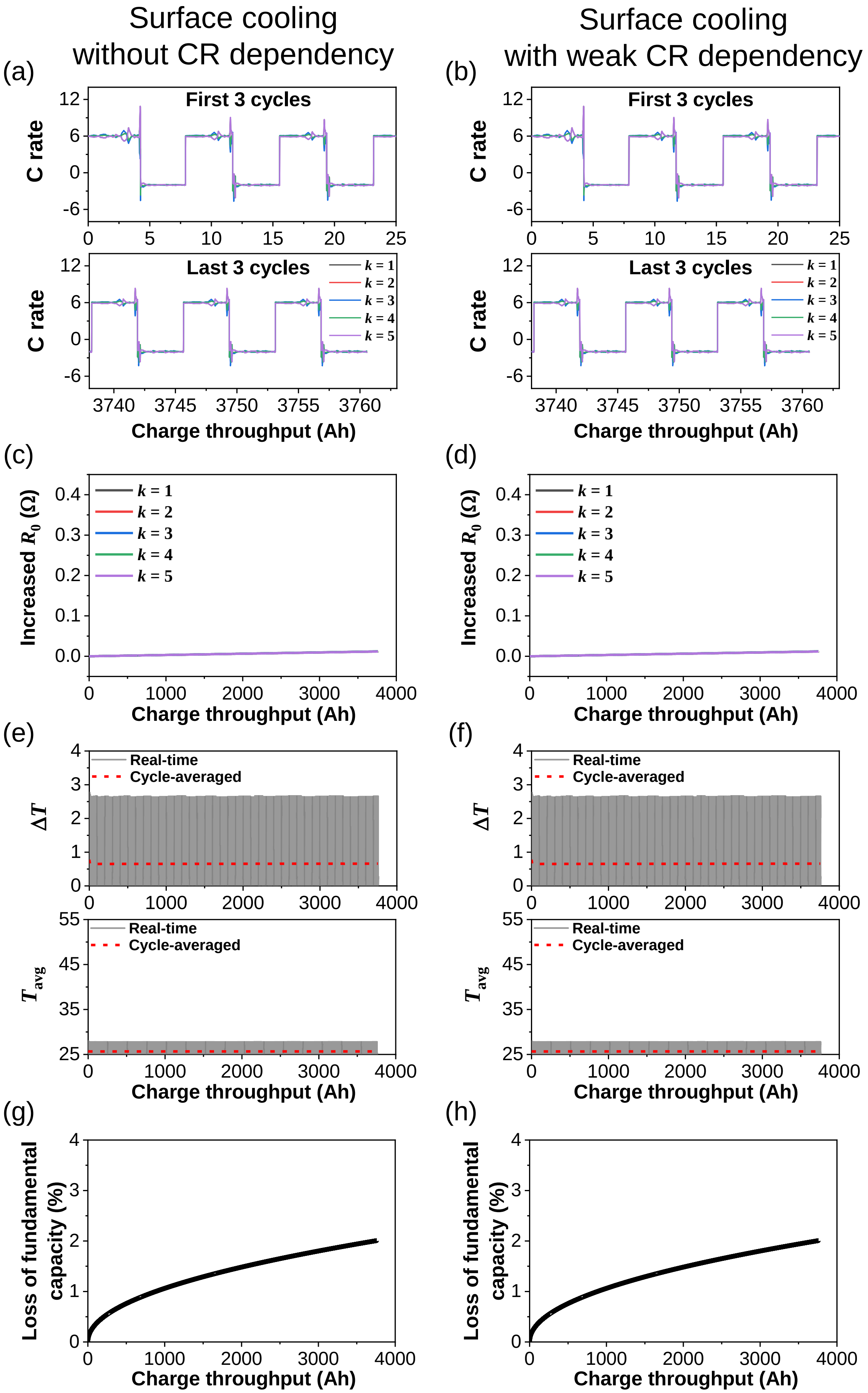
Surface cooling
without CR dependency
Surface cooling
with weak CR dependency
(a)
(b)
First 3 cycles
Last 3 cycles
C rate
k = 1
k = 2
k = 3
k = 4
k = 5
Charge throughput (Ah)
(c)
(d)
Increased R_0 (Ω)
(e)
(f)
Real-time
Cycle-averaged
ΔT
T_{avg}
(g)
(h)
Loss of fundamental capacity (%)

Fig. S6. Cell degradation simulation under surface cooling using ageing law with no current dependency and weak current dependency. Local C rate distribution for the five ECN units along the centre line in the cell thickness direction: (a) no CR dependency and (b) weak CR dependency. The upper and lower insets show the first and last three cycles, respectively, of the 500 cycles modelled. Increased resistance is shown for the ECN units for (c) no CR dependency and (d) weak CR dependency. The variables for units $k = 1$ to $k = 5$ in (c) and (d) are overlapping. The thermal gradient ΔT across the thickness of the cell and the average cell temperature T_{avg} are shown for (e) no CR dependency and (f) weak CR dependency. The grey lines represent the real-time data and the red dotted curves represent the cycle-averaged value. The loss of fundamental capacity percentage for the whole cell is shown for (g) no CR dependency and (h) weak CR dependency.

Supplementary References

[1] Cordoba-Arenas A, Onori S, Guezennec Y, Rizzoni G. Capacity and power fade cycle-life model for plug-in hybrid electric vehicle lithium-ion battery cells containing blended spinel and layered-oxide positive electrodes. J Power Sources 2015;278:473–83. https://doi.org/10.1016/j.jpowsour.2014.12.047.

[2] Hunt IA, Zhao Y, Patel Y, Offer J. Surface Cooling Causes Accelerated Degradation Compared to Tab Cooling for Lithium-Ion Pouch Cells. J Electrochem Soc 2016;163:A1846–52. https://doi.org/10.1149/2.0361609jes.
